# Validity and clinical utility of a wrist-worn device against polysomnography

**DOI:** 10.1371/journal.pone.0330774

**Published:** 2025-09-30

**Authors:** Junwei Guo, Jinmei Luo, Yi Xiao, Rong Huang

**Affiliations:** 1 Department of respiratory and critical care Medicine, Peking Union Medical College Hospital, Chinese Academy of Medical Sciences & Peking Union Medical College, Beijing, China; 2 Department of Pulmonary and Critical Care Medicine, Centre of Respiratory Medicine, National Centre for Respiratory Medicine, State Key Laboratory of Respiratory Health and Multimorbidity, National Clinical Research Centre for Respiratory Diseases, Institute of Respiratory Medicine, Chinese Academy of Medical Sciences, China-Japan Friendship Hospital, Beijing, China; Charité - Universitätsmedizin Berlin, GERMANY

## Abstract

**Objective:**

Sleep-wearable technology has developed rapidly. However, few carried out validation in the real clinical settings. This study aimed to validate the performance of a consumer-grade sleep-tracking device compared to polysomnography (PSG) in participants from sleep clinics.

**Methods:**

Participants referred to sleep clinic from 2021 to 2023 were recruited. Demographics and sleep questionnaires were also collected. All participants completed the PSG test in a sleep laboratory, along with a smart watch (HUAWEI WATCH GT2) that collected movement and heart rate signals using built-in sensors. Epoch-by-epoch agreement analysis and the Bland-Altman method were applied to evaluate the performance of smart watch.

**Results:**

98 participants were included in this study. 82 of them were men, with a mean age of 45.3 ± 10.6 years. The smart watch had a high sensitivity (95.9%), accuracy (87.3%), positive predictive value (72.2%), and relatively low specificity (47.9%) for sleep/wake performance. Sleep staging comparisons were mixed. Comparing to PSG, although smart watch tended to overestimate total sleep time (+28.7 min, P = 0.001), sleep efficiency (+5.94%, P < 0.001), sleep onset latency (+8.53 min, P < 0.001) and underestimate wake after sleep onset (−37.00 min, P < 0.001), acceptable agreement was observed in sleep/wake detection (Kappa coefficient>0.4), total sleep time and sleep efficiency (intraclass correlation coefficient>0.4). This agreement was less satisfactory in patients with OSA or insomnia.

**Conclusion:**

This study compared the performance of a consumer-grade sleep-tracking device with that of PSG. The HUAWEI WATCH GT2 exhibited high agreement in sleep/wake detection. Such devices could be used as alternatives for successive sleep detection and could provide significant benefits to sleep hygiene with more advanced algorithms in the future.

## Introduction

Sleep accounts for one-third of an individual’s life. Poor sleep is associated with various morbid conditions including cardiovascular disease, dementia, and sudden death. In clinical practice, sleep is measured by polysomnography (PSG), a procedure collects multiple biological signals during sleep. Despite being a golden standard technique for sleep-breathing disorders, PSG has several shortcomings that limit its application. These include low cost-effectiveness, first-night effects, night-to-night variability, and the need for professional technicians and doctors. Owing to their considerable cost, sleep centers usually carry out single-night sleep monitoring. Therefore, certain sleep disorders may have been overlooked. Alternative methods for PSG include portable monitoring and actigraphy, which overcome some of these issues but are still imprecise and restricted to certain populations [1].

The concept of wearable technologies was first proposed in the 1960s [2]. Since then, owing to the development of artificial intelligence, sleep-wearable technology, especially in the consumer market, has developed rapidly. Similar to actigraphy, these consumer devices detect an individual’s movements to determine their sleep status using built-in accelerometers. Furthermore, some wearables claim to possess machine learning and use multiple sensors to improve their performance and provide information other than sleep/wake detection. However, these functions have not been fully validated. Private and frequently updated algorithms and rapidly iterative products make it difficult to transform them into clinical practice. In fact, overwhelming messages provided by wearables often frustrate the customers. Some may have unnecessary worries about their situation and seek medical help.

Despite the limitations mentioned above, previous studies have shown that consumer sleep technology (CSTs) have equivalent performance to actigraphy, while other information, such as sleep staging acquired from new sensors, has mixed results [3–5]. Most studies performed validation in healthy subjects, which could not fully represent clinical settings. Several studies have validated patients with sleep disorders such as insomnia and OSA [6–8]. However, these studies simply demonstrated the performance of different devices without further comparison among disease subgroups. Further evidence regarding new functions in CSTs and clinical populations is required.

The most validated commercial devices are from Fitbit. However, since the takeover by Google in 2021, Fitbit devices have possessed less of a market for wearable technologies. Other commercial devices, such as Apple, Samsung, and HUAWEI, are welcomed by consumers but have not been validated by researchers. The disproportionate ratio of market possession to device validation indicates a need for further evaluation. The HUAWEI smart watch dominates the sales share in the Chinese smart watch market [9]. It was claimed that the device could provide various personal health information including sleep, blood glucose, and heart rhythm due to built-in signals such as photoplethysmography, motion, and heart rate variation. A previous study proved its feasibility for atrial fibrillation screening [10], but little is known about its performance in sleep detection.

This study aimed to validate the performance of a commercial smart watch, HUAWEI WATCH GT2, which collects movement and heart rate variation signals for sleep detection, against PSG and compare the performance of a smart watch in clinical settings across participants with different sleep disorders.

## Methods

### Study population

Participants referred to sleep clinic with suspected sleep disorders were recruited from March 1st 2021 to April 30th 2023. The following inclusion criteria were applied: (1) age > 18 years, (2) completion of demographic and sleep questionnaires, and (3) willingness to wear a smart watch while monitoring sleep with PSG. The exclusion criteria were as follows: (1) total sleep time < 4h; (2) other conditions such as traumatic brain injury, dementia, or stroke that would affect their comprehension of informed consent; (3) recent (< 1 month) treatment for sleep disorders such as hypnotics, cognitive behavior therapy for insomnia (CBT-I), or continuous positive airway pressure (CPAP) for OSA; and (4) engaged in shift work within the last 6 months. The study was approved by the ethics committee of Peking Union Medical College Hospital (JS-2089) and was conducted in accordance with the Declaration of Helsinki ethic requirements. All participants provided written consent prior to the study.

### Demographics

Baseline characteristics were collected questionnaires and clinical examinations. Demographics, medical history, medication use, and personal behaviors were recorded. Body mass index (BMI) and waist-to-hip ratio (WHR) were calculated based on the demographics. Self-reported questionnaires, including the Pittsburgh Sleep Quality Index (PSQI), Epworth Sleepiness Scale (ESS), and Insomnia Severity Index (ISI), were used to evaluate the sleep quality, daytime sleepiness, and insomnia severity. Participants with PSQI scores of ≥ 5 were considered to have poor sleep quality. Daytime sleepiness was defined as an ESS score ≥ 10. Clinical insomnia was confirmed if participants claimed to take hypnotics or had an ISI score ≥ 15.

### Overnight polysomnography

All participants completed a single-night PSG (Embla N7000, Natus Medical Incorporated, Orlando, FL, USA) monitoring in the sleep center from 11 p.m. to 6 a.m. A certified sleep laboratory technician scored the sleep stages and respiratory events according to the American Academy of Sleep Medicine (AASM) standard protocol recommendations. Five stages, awake, N1, N2, N3, and rapid eye movement (REM) sleep, were recorded using PSG. Epochs of 30s were used for sleep summary and epoch-by-epoch (EBE) analysis. Apnea was defined as a decrease in respiratory airflow by 90% from baseline for more than 10 seconds. Hypopnea was defined as a decrease in respiratory airflow of 30% for more than 10 s, accompanied by a decrease in oxygen saturation of more than 3%, or arousal. Apnea hypopnea index (AHI) ≥ 15/h was used to diagnose OSA. Other hypoxemia indices, such as the percentage of time spent with SpO_2_ < 90% (T90), oxygen desaturation index (ODI), and lowest pulse oxygen saturation (LSpO_2_) were also collected.

### Smart watch measurements

**Fig 1:**
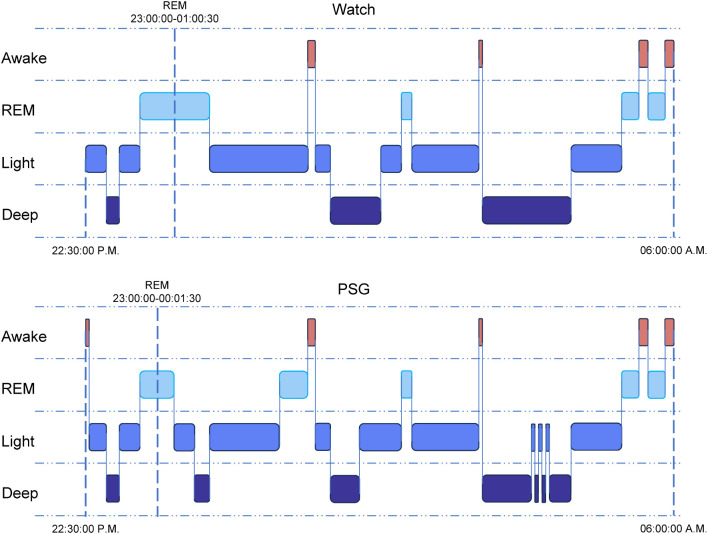
Schematic graph of sleep monitoring by smart watch and PSG. The same lights off (22:00 P.M.) and lights on (06:00 A.M.) time were strictly set. In this example, for smart watch, the time from lights off to the detection of sleep data (22:30:00 P.M.) was recognized as sleep onset latency (SOL, 30 min in this example). 30s of epochs are generated from the summary graphs of smart watch and PSG. The start and end time of every sleep stage could also be obtained from the graph (REM from 23:00:00 to 01:00:30 for smart watch and REM from 23:00:00 to 00:01:30 for PSG in this example). By comparing with PSG, the agreement of each 30-second epoch from the smart watch can be evaluated.

At the beginning of sleep tracking (lights off, 23:00 P.M.), the research staff ensured that the smart watch was correctly worn on the non-dominant wrist. The study design followed a standard framework for testing the performance of CSTs proposed in an earlier study [11]. To ensure the same length of total recording time (TRT) for data synchronization, the watch was removed at the same time when PSG was completed (lights on, 6:00 A.M.). Data for the watch were not available as output files from the software. Accordingly, sleep stage information for each 30s epoch was manually extracted from the summary graphs on the mobile app designed for the research study. Details about this process are presented in Fig 1. The smart watch provides four stages of sleep recordings based on movement and heart rate variation signals: awake, light sleep, deep sleep, and REM sleep. After checking the specifications with the manufacturer, it was confirmed that light sleep equaled stages N1 and N2 and deep sleep equaled stage N3. The measurements of interest, including total sleep time (TST), sleep efficiency (SE, TST/minutes between lights off and lights on), and wake after sleep onset (WASO), were generated according to the scored epochs of the PSG and the smart watch. Sleep latency was calculated in two ways: (1) sleep onset latency (SOL), the time from lights off to the first epoch scored as any sleep stage, and (2) latency to persistent sleep (LPS), the time from lights off to the first epoch of 10 consecutive minutes scored as any sleep stage. The latter is often used to assess the sleep quality of patients with insomnia.

### Statistical analysis

Baseline demographics and sleep measurements are presented as mean (± SD) or median (interquartile range, 25%−75%) for continuous variables, depending on the data distribution. Categorical variables are summarized as frequencies with proportions.

The following EBE agreement statistics were calculated for the sleep/wake and sleep stage analyses: sensitivity, specificity, accuracy, Cohen’s Kappa coefficient, and prevalence and bias-adjusted kappa (PABAK). Matthews correlation coefficient (MCC) was presented to further evaluate robustness of the results. A confusion matrix was used to calculate the true-positive (TPs), true-negative (TNs), false-positive (FPs), and false-negative (FNs) values. The sensitivity, specificity, accuracy, positive predictive value (PPV), and negative predictive value (NPV) were calculated based on the confusion matrix. Cohen’s kappa coefficient was used to calculate the percentage of scoring agreement between the two devices, not due to chance, and PABAK further gave balanced weight to epochs [12]. MCC was calculated using following equation:


$$MCC=\;\frac{TP\times TN-FP\times FN}{\sqrt{\left(TP+FP)\times (TP+FN)\times (TN+FP)\times (TN+FN\right)}}$$

Parameters with a Kappa coefficient > 0.4 was considered acceptable agreement. These metrics were calculated for each subject and averaged to generate group-level EBE performance. Agreement was interpreted according to recommended guidelines: 0–0.20 indicates slight agreement, 0.21–0.40 is fair, 0.41–0.60 is moderate, 0.61–0.80 is substantial, and 0.81–1.0 is almost perfect [13].

Bland-Altman plots were used to assess the agreement between PSG and the smart watch for each continuous sleep parameter. The overall levels of bias and upper and lower limits of agreement are presented. The mean of the two measurements was used to represent the size of the measurements. Sleep summary parameters were also statistically compared with PSG using Student’s paired t-tests, intraclass correlation coefficient (ICC) using the absolute agreement mode, and Hedges’ g effect sizes.

To test whether the smart match had different performances in participants with sleep disorders, the agreement between the two devices in OSA or insomnia group was further compared with that of healthy controls in the second analysis using chi-square test.

All data were analyzed using R software (RStudio running R version 4.2.2). A two-sided P-value of <0.05 was considered significant.

## Results

### Baseline characteristics

**Fig 2:**
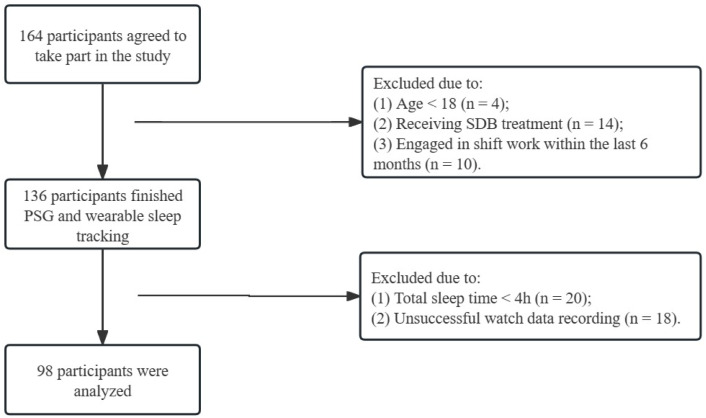
Flowchart of study population.

In total, 164 participants were consecutively recruited for this study. 66 were excluded due to participants’ condition or data loss. 98 met the inclusion criteria and were analyzed finally. Fig 2 showed population recruitment of this study. Among these participants, 82 (83.7%) were male with a mean age of 45 years and a mean BMI of 26.0 kg/m^2^. More than 50% of participants complained of poor sleep quality and daytime sleepiness. The median TST and SE were 405.8 min and 85%, respectively. According to the PSG results, 33 patients were normal participants and 47 had moderate-to-severe OSA (AHI > 15/h). 30 were considered to have clinical insomnia with an ISI ≥ 15. 12 were suffered from comorbid insomnia and sleep apnea. The baseline patient characteristics are listed in Table 1.

**Table 1:** Baseline demographic and sleep parameters.

Variables	Overall (n = 98)
**Age, y**	45.3 ± 10.6
**Sex, male, n (%)**	82 (83.7)
**Race, Han, n (%)**	93 (94.9)
**WHR, %**	0.94 ± 0.06
**BMI, kg/m** ^**2**^	26.0 (24.7-28.7)
**Hypertension, n (%)**	26 (27.7)
**CVD, n (%)**	12 (12.8)
**Smoke, n (%)**	31 (31.6)
**Alcohol use, n (%)**	78 (79.6)
**Hypnotic use, n (%)**	7 (7.14)
**ESS > 10, n (%)**	54 (55.1)
**PSQI ≥ 5, n (%)**	84 (85.7)
**ISI ≥ 15, n (%)**	25 (25.5)
**TST, min**	405.8 (364.5-433.0)
**Sleep efficiency**	85.0 (76.5-91.7)
**AHI,/h**	14.4 (5.1-33.5)
**ODI,/h**	10.6 (3.9-28.0)
**LSpO**_**2**_ **, %**	88.0 (84.0-91.0)
**T90, %**	0 (0-0.3)

WHR, waist hip ratio; BMI, body mass index; CVD, cardiovascular disease; ESS, Epworth Sleepiness Scale; PSQI, Pittsburgh Sleep Quality Index; ISI, insomnia severity index; TST, total sleep time; AHI, apnea-hypopnea index; ODI, oxygen desaturation index; LSpO2, lowest pulse oxygen saturation; T90, timespent with SpO2 < 90%.

### Overall performances of smart watch against PSG

**Fig 3:**
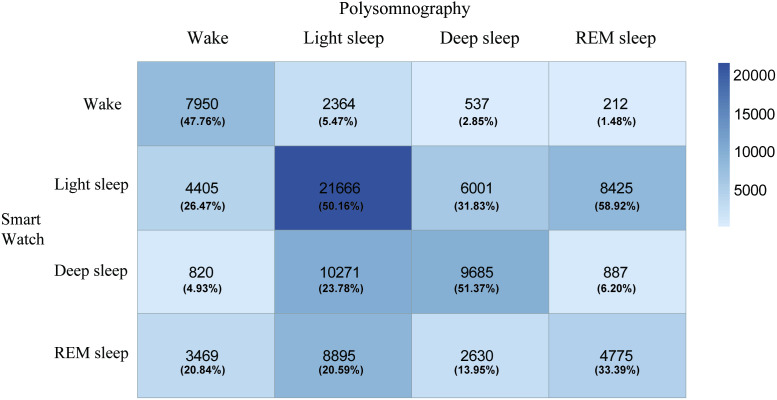
Confusion matrix of sleep staging agreement between polysomnography and smart watch. The percentages of each sleep stage scored by smart watch are presented. Deeper color indicates higher frequencies.

In the final analyses, 92992 epochs were exported manually for comparison. A confusion matrix illustrating the sleep staging agreement between the PSG and smart watch is presented in Fig 3. Overall, the PSG and smart watches had higher agreement in the wake and light sleep classifications. There were high error rates for the smart watch in misclassifying PSG REM epochs as light sleep. Similarly, for PSG-scored epochs that differed from the device, device epochs scored as deep sleep and REM sleep were often classified as light sleep. Misclassification errors among other possible stage classifications were comparatively low.

For EBE agreement of sleep versus wake state compared with PSG, the smart watch had a high sensitivity of 95.3% and a low specificity of 44.5%. The PPV was 72.20% and the kappa coefficient reached acceptable agreement (κ = 0.43) between the two devices. After adjusting for the prevalence and bias of epochs, the PABAK improved substantially (κ = 0.75). For sleep staging agreement, smart watch showed high accuracy (>70%) for all sleep stages with the exception of light sleep. The PABAK coefficients implied moderate to substantial agreement between the PSG and smart watch for deep and REM sleep. MCC reached acceptable agreement (0.48) for sleep/wake classification which was highest among sleep stages detection. (Table 2).

**Table 2:** Summary of epoch-by-epoch analysis for smart watch against PSG.

Sleep stage	Sensitivity (%)	Specificity (%)	Accuracy (%)	PPV (%)	NPV (%)	Kappa (κ)	PABAK	MCC
**Wake**	44.46 (31.90) [38.20-50.75]	95.34 (10.41) [93.49-97.54]	87.35 (12.14) [85.11-89.85]	30.55 (17.63) [26.94, 34.12]	45.61 (17.36) [43.33, 49.52]	0.43 (0.29) [0.38-0.49]	0.75 (0.24) [0.70-0.80]	0.48 (0.28) [0.43-0.53]
**Sleep**	95.34 (10.41) [93.46, 97.55]	44.46 (31.90) [38.16, 50.88]	87.35 (12.14) [85.09, 89.89]	72.20 (17.08) [68.79, 75.55]	47.82 (19.66) [43.67, 51.45]	0.43 (0.29) [0.38, 0.49]	0.75 (0.24) [0.70-0.80]	0.48 (0.28) [0.43-0.53]
**Light**	50.09 (13.40) [47.41-52.82]	62.21 (10.89) [60.09-64.35]	56.61 (8.55) [54.96-58.27]	45.23 (8.40) [43.61, 46.88]	49.76 (0.54) [49.67, 49.88]	0.12 (0.16) [0.08-0.15]	0.13 (0.17) [0.10-0.17]	0.12 (0.17) [0.09-0.15]
**Deep**	52.69 (23.18) [48.20-57.32]	84.35 (8.98) [82.58-86.12]	77.20 (8.15) [75.62-78.82]	38.72 (13.70) [36.11, 41.50]	47.65 (4.71) [46.87, 48.71]	0.32 (0.20) [0.28-0.36]	0.54 (0.16) [0.51-0.58]	0.34 (0.21) [0.30-0.38]
**REM**	34.50 (18.60) [30.82-38.12]	80.97 (7.04) [79.60-82.37]	73.66 (6.97) [72.30-75.01]	30.23 (12.74) [27.77, 32.74]	48.68 (5.58) [47.38, 49.53]	0.12 (0.15) [0.09-0.15]	0.47 (0.14) [0.45-0.50]	0.13 (0.16) [0.10-0.16]

PPV, positive predictive value; NPV, negative predictive value; PABAK, prevalence and bias-adjusted kappa; MCC, Matthews correlation coefficient.

Data are reported as the mean (standard deviation) [95% confidence intervals].

**Fig 4:**
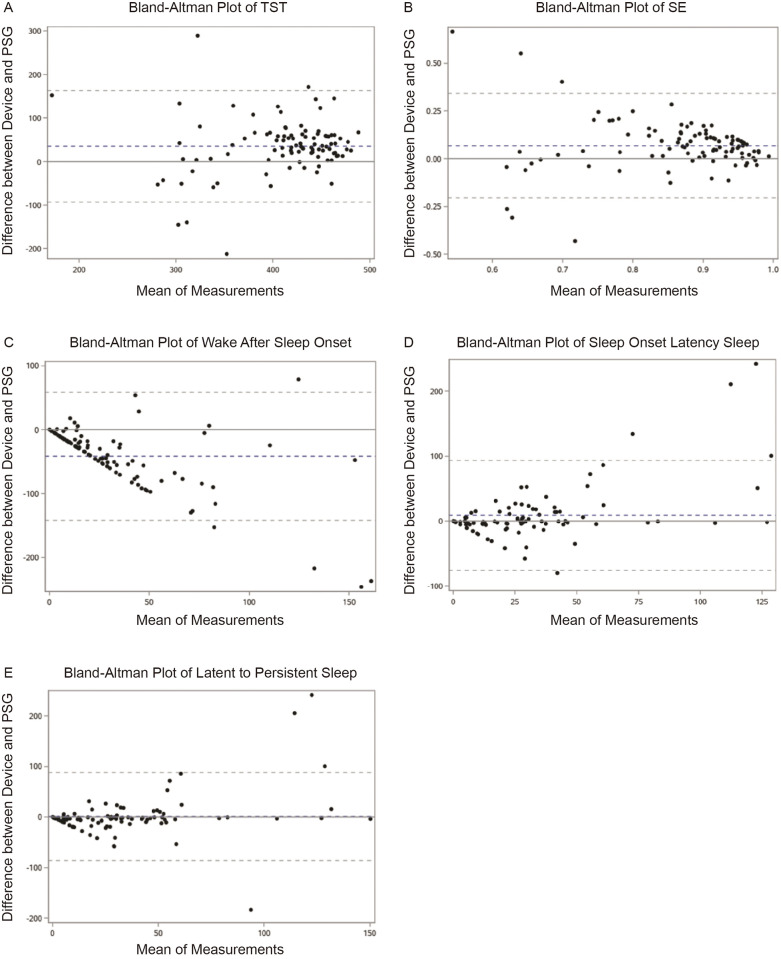
Bland-Altman plot between polysomnography and smart watch. Mean bias, upper and lower limits of agreement are presented. (A) total sleep time, (B) sleep efficiency, (C) wake after sleep onset, (D) sleep onset latency, (E) latency to persistent sleep.

For continuous sleep summary results (Table 3), according to mean bias values and paired t-tests, except for light sleep (P = 0.07), smart watch significantly overestimated TST, SE, SOL, deep sleep, and REM sleep by 28.5 min (P < 0.001), 5.94% (P < 0.001), 8.53 min (P = 0.048), 14.3 min (P = 0.038), and 27.91 min (P < 0.001), respectively, while underestimated WASO by 37 min (P < 0.001). After adjusting for unstable sleep, the LPS levels between the two devices were not significantly different (P = 0.812). Bland-Altman plots comparing the smart watch with PSG for each sleep variable are presented in Fig 4. Biases were generally the lowest magnitude and least variable when participants had higher TST and SE, and were more variable and biased in participants with lower TST and SE. In contrast, for individuals with lower WASO/SOL (and thus higher TST and SE), the agreement between the smart watch and PSG was better, and when there was higher WASO/SOL, the differences were more variable. Similarly, Bland-Altman plots showed better agreement with LPS comparing to SOL.

**Table 3:** Summary of sleep indices for the smart watch against PSG.

Measures	Device Mean±SD	PSG Mean±SD	Bias Mean (95% CI)	t (P)	ICC (P)	Effect sizes (Hedges’ g)
**TST**	418.01 ± 64.85	389.53 ± 66.25	28.47 (15.01-41.94)	4.20 (< 0.001)	0.437 (< 0.001)	0.433
**N1-2**	206.62 ± 39.75	220.39 ± 73.22	−13.77 (−28.77-1.23)	−1.82 (0.072)	0.190 (0.028)	−0.233
**N3**	110.53 ± 39.82	96.19 ± 53.71	14.34 (0.80-27.87)	2.10 (0.0381)	0.019 (0.577)	0.302
**REM**	100.86 ± 32.99	72.95 ± 27.71	27.91 (19.52-36.30)	6.60 (< 0.001)	0.040 (0.288)	0.912
**SE (%)**	88.17 ± 13.31	82.23 ± 14.02	5.94 (3.14-8.74)	4.21 (< 0.001)	0.439 (< 0.001)	0.433
**WASO**	22.11 ± 40.17	59.11 ± 60.83	−37.00 (−47.44--26.56)	−7.03 (< 0.001)	0.391 (< 0.001)	−0.715
**SOL**	34.33 ± 45.08	25.81 ± 24.85	8.53 (0.08-16.97)	2.00 (0.048)	0.324 (< 0.001)	0.233
**LPS**	34.39 ± 45.08	33.34 ± 32.38	1.05 (−7.69-9.78)	0.24 (0.812)	0.386 (< 0.001)	0.027

TST, total sleep time; REM, rapid eye movement; SE, sleep efficiency; WASO, wake after sleep onset; SOL, sleep onset latency; LPS, latency to persistent sleep; ICC, interclass correlation coefficient.

### Performances in patients with sleep disorders

Subgroup analysis was performed to validate the performance of the smart watch in participants with certain sleep disorders. The performance in healthy controls showed the best agreement in sleep/wake detection among all participants, with the highest values of sensitivity (97.4%), accuracy (89.9%), kappa (κ = 0.46), PABAK coefficient (κ = 0.80), and MCC (0.51). Compared to healthy controls, t-tests revealed lower accuracy in patients with insomnia (83.0%, P = 0.038) and lower sensitivity in patients with OSA (92.5%, P = 0.028), suggesting relatively poor performance of the smart watch in patients with sleep disorders (Table 4). No significant differences were observed in sleep staging agreement among the different sleep disorders. For continuous sleep summary results, the smart watch showed similar performance across subgroups, with overestimation of TST, SE, and REM sleep and underestimation of WASO (Table 5).

**Table 4:** Epoch by epoch agreement for smart watch against PSG for sleep detecting in patients with sleep disordered breathing.

Status	Sensitivity (%)	Specificity (%)	Accuracy (%)	Kappa (κ)	PABAK	MCC
**Normal**	97.36 (96.03-98.87)	45.72 (34.35-56.91)	89.91 (87.54-92.65)	0.46 (0.36-0.56)	0.80 (0.75-0.85)	0.51 (0.41-0.61)
**Insomnia**	94.19 (90.39-99.1)	37.59 (25.57-48.89)	83.03 (77.85-89.05) ^#^	0.35 (0.25-0.45)	0.66 (0.56-0.78)	0.41 (0.31-0.50)
**OSA**	92.45 (88.83-96.79) ^#^	48.95 (40.06-57.49)	85.48 (81.77-89.61)	0.44 (0.36-0.52)	0.71 (0.64-0.79)	0.48 (0.41-0.56)
**All**	95.34 (93.49-97.54)	44.46 (38.20-50.75)	87.35 (85.11-89.85)	0.43 (0.38-0.49)	0.75 (0.70-0.80)	0.48 (0.43-0.53)

OSA, obstructive sleep apnea; PABAK, prevalence and bias-adjusted kappa; MCC, Matthews correlation coefficient.

Comparisons were made between insomnia/OSA and healthy control.

Data are reported as the mean (95% confidence intervals).

# P < 0.05.

**Table 5:** Summary of sleep indices for the smart watch against PSG.

Sleep stage	Status	Sensitivity (%)	Specificity (%)	Accuracy (%)	Kappa (κ)	PABAK	MCC
Light	Normal	47.74 (10.75) [44.12 - 51.31]	62.22 (9.77) [58.98 - 65.46]	56.63 (7.72) [54.05 - 59.24]	0.10 (0.13) [0.05 - 0.14]	0.13 (0.15) [0.08 - 0.18]	0.10 (0.14) [0.05-0.14]
	Insomnia	51.32 (13.59) [46.56 - 56.12]	61.33 (12.62) [56.83 - 65.76]	56.63 (9.45) [53.28 - 59.97]	0.12 (0.18) [0.06 - 0.19]	0.13 (0.19) [0.06 - 0.20]	0.13 (0.19) [0.06-0.19]
	OSA	52.14 (15.15) [47.99 - 56.55]	63.67 (11.38) [60.46 - 66.78]	57.46 (8.92) [54.96 - 59.92]	0.15 (0.17) [0.10 - 0.20]	0.15 (0.18) [0.10 - 0.20]	0.16 (0.18) [0.10-0.21]
Deep	Normal	56.97 (20.57) [50.14, 63.97]	84.03 (8.81) [81.07, 87.02]	77.08 (6.29) [75.00, 79.24]	0.37 (0.18) [0.30, 0.43]	0.54 (0.13) [0.50, 0.59]	0.39 (0.18) [0.33-0.45]
	Insomnia	49.34 (23.42) [41.5, 57.83]	84.67 (8.52) [81.71, 87.67]	76.98 (9.52) [73.74, 80.38]	0.30 (0.20) [0.23, 0.37]	0.54 (0.19) [0.47, 0.61]	0.31 (0.21) [0.24-0.39]
	OSA	48.11 (25.69) [41.06, 55.57]	85.30 (9.28) [82.74, 87.98]	77.98 (9.25) [75.37, 80.60]	0.28 (0.22) [0.22, 0.34]	0.56 (0.18) [0.51, 0.61]	0.30 (0.23) [0.24-0.37]
Rem	Normal	33.86 (15.26) [28.75, 38.99]	81.15 (6.99) [78.88, 83.55]	73.02 (6.68) [70.82, 75.32]	0.13 (0.12) [0.09, 0.17]	0.46 (0.13) [0.42, 0.51]	0.13 (0.13) [0.09-0.18]
	Insomnia	31.99 (19.97) [24.93, 39.02]	80.08 (6.63) [77.69, 82.37]	72.50 (6.78) [70.08, 74.94]	0.09 (0.16) [0.03, 0.14]	0.45 (0.14) [0.40, 0.50]	0.09 (0.17) [0.03-0.15]
	OSA	35.71 (20.27) [29.80, 41.30]	82.11 (7.34) [80.01, 84.15]	75.31 (7.11) [73.32, 77.31]	0.13 (0.16) [0.09, 0.18]	0.51 (0.14) [0.47, 0.55]	0.15 (0.16) [0.10-0.19]

Reported as mean (standard deviation) [95% confidence intervals].

PABAK, prevalence and bias-adjusted kappa; MCC, Matthews correlation coefficient.

Data are reported as the mean (standard deviation) [95% confidence intervals].

## Discussion

This study performed a sleep validation test against PSG for HUAWEI WATCH GT2, a smart wrist-worn consumer device with multiple healthcare functions. The test was conducted in a real medical environment to evaluate its clinical feasibility. The results indicated high sensitivity but relatively low specificity and a substantial range of inter-rater reliability for sleep/wake state detection, while the agreement was inconsistent for sleep staging. The device showed a tendency to overestimate the TST and SE and underestimate the WASO. For patients with OSA or insomnia, agreement was less satisfactory. Despite unsatisfactory results in certain aspects, the smart watch can be used as an alternative option for sleep/wake state detection in the general population.

To the best of our knowledge, this is the first validation study on sleep tracking using the HUAWEI WATCH in a Chinese population. The robust result of the agreement in sleep/wake detection indicated its potential usability in daily monitoring. With the rapid development of modern society, more people are suffering from physical and mental illnesses. These side effects often lead to sleep deprivation and disturbances, which perpetuate an individual’s poor condition. The related disease burden has resulted in huge costs to society and families. This urgent situation demands more public awareness of sleep health in the general population.

Previous studies have emphasized multiple dimensions to achieve sleep health [14]. These included the sleep duration, SE, sleep timing, alertness, and sleep satisfaction. Although PSG and clinical questionnaires are standard criteria for sleep assessment, limited medical resources and the recent Covid-19 pandemic have forced us to develop simpler and more accessible modes of sleep monitoring. Single night monitoring by PSG is also difficult for clinicians to evaluate sleep quality over a period of time. Wearables and nearables were developed to overcome these limitations. Compared to PSG, wearables and nearables have millions of customers and do not require medical technicians. In addition, these devices are convenient for monitoring multi-night sleep. Compared to nearables such as mattresses or radar, wearables can directly collect signals from users, regardless of their bed partners. Together with other advantages, such as cellphone messages and GPS location, wearables, especially wrist watches or bands, are welcomed by consumers worldwide.

In contrast to the prosperity of the consumer market, the validation of these devices is scarce. Despite their nonclinical utilization, many still claim sleep detection without user classification, which makes validations of these devices necessary in clinical settings. Many studies have attempted to achieve this by directly comparing parameters such as the TST, SE, and WASO [15–17]. The result could be misinterpreted as an incorrect match between wearables and PSG within a given time while presenting similar parameters. Epoch-by-epoch analysis, as the standard method for validation studies, has been less frequently adopted because of its time-consuming nature and data privacy. This study used this standard method and fully evaluated its feasibility, providing convincing evidence that the HUAWEI WATCH GT2 could serve as an alternative to PSG in the general population.

Many of the current findings on the overall performance are consistent with studies that have also tested the performance of consumer sleep-tracking devices against PSG. For sleep/wake detection, previous studies reported a sensitivity greater than 90%, and specificity ranging from 20% to 80% [18]. The smart watch also showed high sensitivity at the cost of relatively low specificity and overestimated the TST while underestimating the WASO. For the sleep staging results, based on the PABAK coefficient, the smart watch showed a moderate to substantial range of agreement, except for light sleep. High levels of specificity and accuracy were observed for awake, deep, and REM sleep. Disagreements regarding light sleep have also been found in a previous Fitbit validation study [19].

For participants with certain sleep disorders, smart watch performance was less satisfactory. When restricted to healthy participants, the smart watch had the highest sensitivity, accuracy, PABAK coefficients, and MCC. Previous studies have reported similar results in participants with sleep disorders or special occupations, such as police officers and paramedics undergoing shift work [7,19–21]. This poor performance is largely due to the use of activity trackers for sleep/wake scoring. In situation where participants lie motionless yet awake, the tracker may misclassify these non‐movement periods as “sleep” [22]. Such cases are more frequently observed in patients with disturbed sleep, such as those with OSA or insomnia, and are impractical for clinical use. A recent trial also showed that consumer wearables were less accurate for fragmented or disturbed sleep [23].

Based on these results, could the device be used in clinical settings? Currently, there are no criteria for portable devices to judge the degree of agreement that is sufficient for clinical use. Some studies used the following criteria to judge satisfactory agreement by differences of ≤30 min and ≤5% between the devices and PSG for the TST and SE values, respectively [24,25]. Others have directly compared the agreement of devices with actigraphy and tested their substitutability. The smart watch almost reached these criteria and could serve as a low-cost substitute for actigraphy for sleep/wake state detection in a healthy population. However, the inconsistent performance of the participants with sleep disturbances requires further validation. Note the chance-corrected agreements between expert sleep scorers for independently scoring a common set of PSG records using a five-stage categorization of sleep were 0.70, 0.24, 0.57, 0.57, and 0.69 for the W, N1, N2, N3, and R stages, respectively [26]. The low inter-rater reliability of sleep staging in PSG also required the criteria of satisfied sleep staging agreement for evaluating sleep-tracking devices. The smart watch also failed to record 18 participants due to the following reasons: most (n = 10) removed or loosened watch band during sleep monitoring because of uncomfortable experience. Another reason was the shifted position due to major body movements (n = 6). Only few unsuccessful recordings were caused by the device (low battery, n = 1; lost not stored at cloud, n = 1). These reasons indicate improvements of hardware are needed.

This study had several limitations. The study was conducted at a single center with a limited number of participants. Other sleep and mental disorders, including periodic limb movement disorders, narcolepsy, and depression, were not evaluated. The real agreement of smart watches in the healthy controls in this study might be better. Actigraphy was not used as a reference in this study, and a direct comparison between the smart watch and actigraphy was not achieved. However, based on previous studies, the smart watch reached a similar agreement with research-grade actigraphy. Finally, the exact algorithm used in the device is unknown. Iterative generations and products with updated algorithms demand new validations, whereas validated products have become old-fashioned. This issue inhibits the clinical application of wearable consumer devices.

## Conclusions

In summary, the performance of smart watch in sleep/wake detection is comparable to that of research-grade sleep wearables such as actigraphy. In situations where polysomnography is impractical, a smart watch is a reasonable method for estimating 2-stage sleep quality. However, both clinicians and consumers should be aware of sleep stage overestimations and underestimations, and pay attention when interpreting sleep stage information or sleep quality of sleep-disturbed patients. Further studies are required to develop more advanced algorithms for consecutive monitoring.
